# Signal enhancement in cantilever magnetometry based on a co-resonantly coupled sensor

**DOI:** 10.3762/bjnano.7.96

**Published:** 2016-07-18

**Authors:** Julia Körner, Christopher F Reiche, Thomas Gemming, Bernd Büchner, Gerald Gerlach, Thomas Mühl

**Affiliations:** 1 Leibniz Institute for Solid State and Materials Research IFW Dresden, Helmholtzstr. 20, 01069 Dresden, Germany; 2 Institut für Festkörperphysik, Technische Universität Dresden, 01062 Dresden, Germany; 3 Institut für Festkörperelektronik, Technische Universität Dresden, 01062 Dresden, Germany

**Keywords:** cantilever magnetometry, coupled oscillator, iron-filled carbon nanotube, magnetometry, signal enhancement

## Abstract

Cantilever magnetometry is a measurement technique used to study magnetic nanoparticles. With decreasing sample size, the signal strength is significantly reduced, requiring advances of the technique. Ultrathin and slender cantilevers can address this challenge but lead to increased complexity of detection. We present an approach based on the co-resonant coupling of a micro- and a nanometer-sized cantilever. Via matching of the resonance frequencies of the two subsystems we induce a strong interplay between the oscillations of the two cantilevers, allowing for a detection of interactions between the sensitive nanocantilever and external influences in the amplitude response curve of the microcantilever. In our magnetometry experiment we used an iron-filled carbon nanotube acting simultaneously as nanocantilever and magnetic sample. Measurements revealed an enhancement of the commonly used frequency shift signal by five orders of magnitude compared to conventional cantilever magnetometry experiments with similar nanomagnets. With this experiment we do not only demonstrate the functionality of our sensor design but also its potential for very sensitive magnetometry measurements while maintaining a facile oscillation detection with a conventional microcantilever setup.

## Introduction

Over the last decade, magnetic objects of micro- and nanometer size have come into focus of researchers, since they offer a wide range of possible applications. These include magnetic storage techniques and spintronics [1], as well as the study of magnetic microorganisms in biology, for example for applications in hypothermia treatment [2]. A technique to investigate such magnetic particles and samples is cantilever magnetometry. The measurement setup is based on a cantilever oscillating at or close to its resonance frequency, with the sample placed at the free end of the cantilever. When an external magnetic field is applied to the setup, the magnetic interaction of the sample with the field alters the resonance frequency of the cantilever by creating a torque [3]. The resulting frequency shift can be used as measurement signal to derive information on the properties of the sample. In most cases the motion of the cantilever is detected optically, for example via laser deflection or laser interferometry [4]. With decreasing sample size, the cantilever has to be adapted to compensate the weaker magnetic interaction and, therefore, the loss in signal strength of the frequency shift. This is usually achieved through geometric changes, making the cantilever itself very small and thin.

An oscillating cantilever beam can be represented by a harmonic oscillator model for each flexural eigenmode of the beam [5]. Considering an external force gradient as an additional spring constant Δ*k*, the eigenfrequency of the cantilever as a harmonic oscillator is given by:

[1]
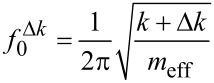


with the spring constant *k* and effective mass *m*_eff_ of the cantilever. Please note that the eigenfrequency *f*_0_ and the resonance frequency 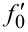 of a harmonic oscillator should in principle be distinguished. They are connected by the relation 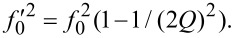 However, it is obvious that in the limit of small dissipation or correspondingly a high quality factor of the cantilever these frequencies coincide well. The resonance frequency of a beam can easily be determined from amplitude response curves and, since all our discussions will be based on such curves, we will be using the term resonance frequency throughout the remainder of this publication.

For magnetometry experiments, the mass of the cantilever with the magnetic sample remains unchanged throughout the experiment, making it unnecessary to consider the influence of mass changes on the resonance frequency of the cantilever. The magnetic interaction between sample and external magnetic field acts as the additional spring constant Δ*k*, altering the resonance frequency of the cantilever. The frequency shift Δ*f* induced by these interactions can be derived from Equation 1 as:

[2]
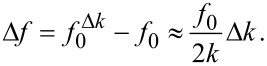


In the case of cantilever magnetometry, Δ*k* does not only depend on the interaction of the magnetic moment *m* of the sample with the external magnetic field *H* but also on the effective length of the cantilever *L*_eff_ [6–7]. Furthermore, by assuming a simple Stoner–Wohlfarth single domain particle, the magnetic interaction is related to the anisotropy field of the sample *H*_a_, so Equation 2 reads:

[3]
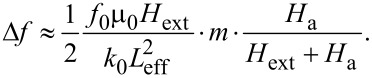


This equation can be used as a good approximation for the iron-filled carbon nanotube samples which are presented in this publication. However, please note that other samples might exhibit more complex magnetic configurations, as for example multiple particles or domains, which then need a more elaborate description.

From Equation 3 it is evident that small magnetic samples require a low stiffness as well as a short length of the cantilever which can be achieved by decreasing all of the dimensions of the cantilever as already mentioned above. As an instructive example to understand this, a simple cantilever with constant rectangular cross section with the width *w*, the thickness *t* and the length *L* can be considered. According to Euler–Bernoulli beam theory, the spring constant and resonance frequency for this kind of beam are given by 
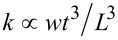
 and 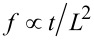
[6]. By combining this with Equation 3 for the frequency shift signal in cantilever magnetometry, it can be derived that 
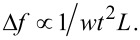
 Therefore, a decrease of all dimensions of the rectangular cantilever is favorable to increase the signal strength. Please note that while this simple derivation is only valid for cantilevers with rectangular cross section, similar considerations can be applied to other cantilever geometries.

However, ultrathin and small cantilevers are difficult to produce and handle and furthermore still need a feature to allow for the use of optical detection methods. This is usually realized by a paddle-shaped structure positioned somewhere along the length of the cantilever [4,8]. Still, the detection capability limits the decrease in size. Conclusively, there are two competing conditions: on the one hand, the stiffness and length of the cantilever should be very low in order to obtain a strong signal even with very small magnetic samples. On the other hand, detection becomes increasingly difficult when the size of the cantilever is reduced to dimensions on the nanoscale.

Our recently introduced sensor concept addresses these difficulties by co-resonant coupling of a micro- and a nanocantilever where the latter allows for very high sensitivity and the micrometer size part for an easy detection [9]. We will only briefly review the theoretical considerations regarding the sensor concept since it is discussed in depth elsewhere [9]. The main focus of this publication is to demonstrate the applicability of the concept for cantilever magnetometry by deriving magnetic information of an iron nanowire and comparing them to the results of other measurements. In our experiment we use a commercially available silicon cantilever of micrometer dimensions and an iron-filled carbon nanotube (FeCNT), the latter with two out of three dimensions on the nanometer scale and therefore with low stiffness and low effective mass. Typical diameters of the iron filling are (15–30) nm and the nanotube length ranges from (15–45) μm [10–11]. The FeCNT not only features favorable geometric and material properties [12] but the iron filling allows for a magnetometry measurement as well and, therefore, for a demonstration of the functionality of our sensor. The remainder of this publication is structured as follows: first we will introduce the main ideas of the co-resonant sensor concept. Next, we will discuss the fabrication of such a sensor and then evaluate a magnetometry measurement and derive magnetic properties of an iron nanowire in order to prove the applicability of the concept and to indicate its potential for signal enhancement in magnetometry.

## Experimental

### Co-resonant concept

**Figure 1:**
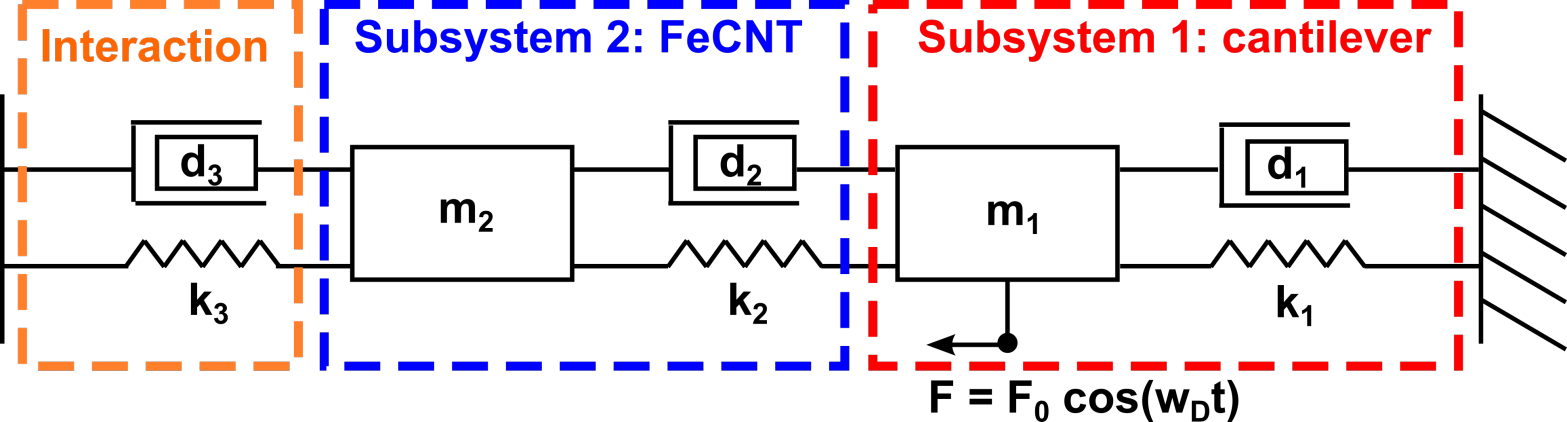
Simple model for two coupled harmonic oscillators, each represented by a mass (*m*_1_, *m*_2_), a sping (*k*_1_, *k*_2_) and a damping element (*d*_1_, *d*_2_). The system is excited to oscillations by a periodic force with the driving angular frequency ω_D_ = 2π*f*_D_ applied to the first subsystem. Interactions between the system and external influences are modeled by an additional spring *k*_3_ and the damping element *d*_3_. For the described sensor setup, subsystem 1 corresponds to the cantilever and subsystem 2 to a FeCNT.

By applying the harmonic oscillator model for both subsystems of our sensor approach, the simple model of a coupled harmonic oscillating system is derived as depicted in Figure 1. It consists of a spring, a mass and a damping element for each subsystem. Furthermore, there are an additional spring *k*_3_ and a damping element *d*_3_, modeling interactions between the coupled system and external influences. The oscillation of the coupled system is driven by a periodic force applied to the bigger subsystem.

**Figure 2:**
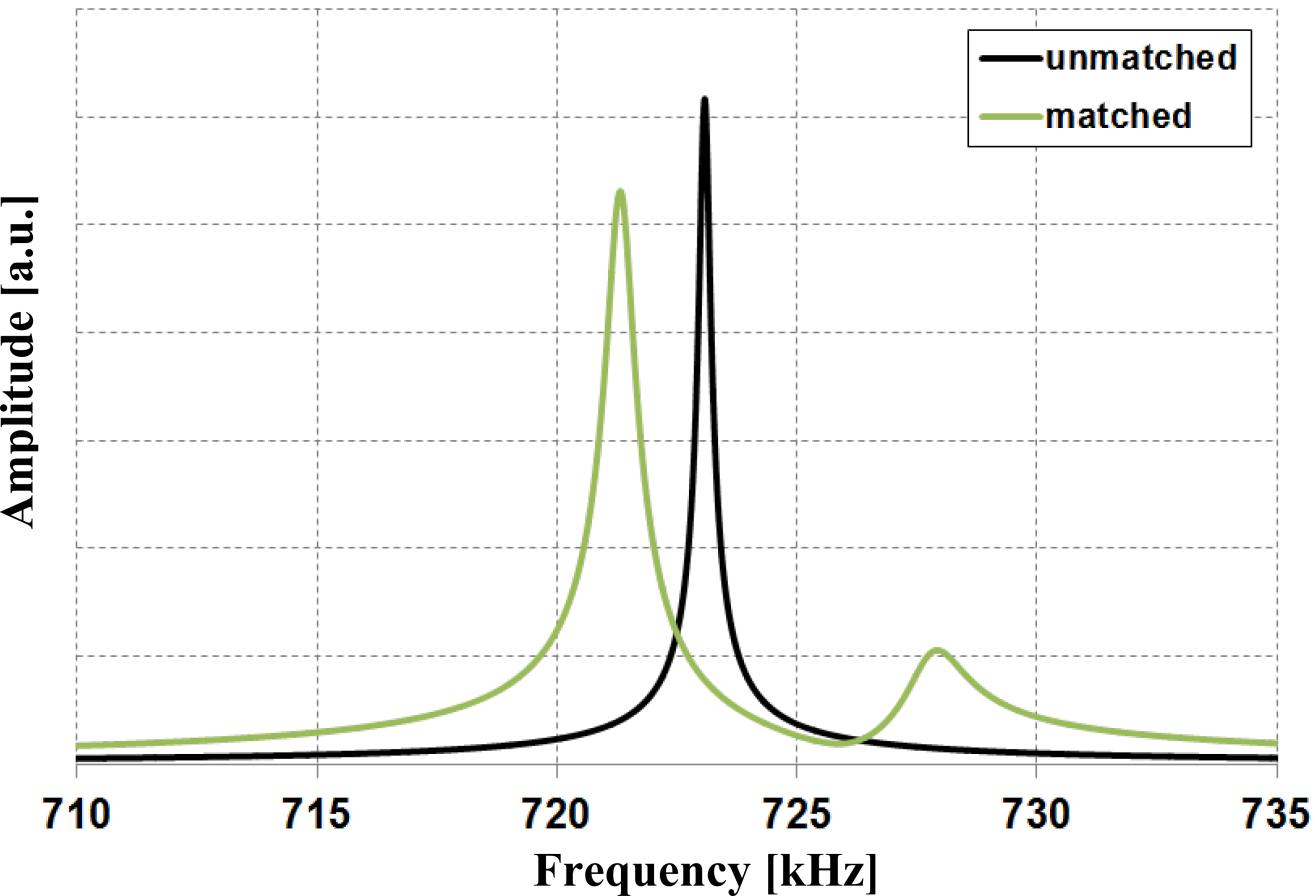
Calculated amplitude response for the cantilever (subsystem 1) with and without frequency matching between the two subsystems. The calculation is based on typical values for cantilever and FeCNT (see Table 1) and *d*_3_ and *k*_3_ are set to zero, assuming a system without interaction.

In our case, subsystem 1 represents the silicon cantilever and subsystem 2 the FeCNT and, since the cantilever is the part of the sensor that will be used for detection, we will discuss the behaviour of the coupled system accordingly. Please note that all the following considerations are valid for the FeCNT as well. Theoretically, if coupled beams are each represented by a harmonic oscillator model which is only valid for one resonance mode of the beam, the amplitude response of each subsystem should exhibit a number of resonance frequencies according to the number of subsystems. Applied to our system it means that two resonance peaks should occur in the amplitude response of the subsystem representing the cantilever. However, if the resonance frequencies of cantilever and FeCNT are far apart, the amplitude of the second peak in the amplitude response of the cantilever will be well below the limit of almost every detection method. Figure 2 depicts a calculated amplitude response of the cantilever based on typical values for our system as summarized in Table 1. With the resonance frequencies of the subsystem far apart, i.e., very weak interplay, the amplitude response curve of the harmonic oscillator representing the cantilever only shows one prominent resonance peak, coinciding well with the resonance frequency of the uncoupled cantilever. The amplitude for the second peak caused by the FeCNT is by several orders of magnitude smaller and therefore not shown in Figure 2.

**Table 1:** Properties of cantilever and FeCNT constituting the coupled sensor.

Parameter	Cantilever (1)	FeCNT (2)

spring constant *k*_i_	133.8 N/m	0.0086 N/m
effective mass *m*_eff,i_	6.5 · 10^−12^ kg	4.1 · 10^−16^ kg
quality factor *Q*_i_	3390	450
resonance frequency *f*_i_ before matching	723080 Hz	2082080 Hz
resonance frequency *f*_i_ after matching	723080 Hz	725610 Hz

This picture changes drastically when the resonance frequency of the FeCNT is adjusted close to the value of the cantilever. In that case, we observe two clear resonance peaks in the amplitude response of the cantilever (Figure 2). Furthermore, due to strong interplay between the subsystems induced by the co-resonant frequency matching, the two resonance frequencies of the coupled system are shifted compared to the resonance frequencies of the single subsystems. We will therefore use *f*_a_ and *f*_b_ for the resonance frequencies of the coupled system and *f*_1_ and *f*_2_ for the individual resonance frequencies of cantilever and FeCNT, respectively, in the following. Futher details on the behaviour of a co-resonantly coupled system can be found elsewhere [9], so we will only summarize the main points here:

1. A strong interplay is induced between the two subsystems due to the matching of the resonance frequencies, even for two highly asymmetric subsystems.
2. Interactions between FeCNT and an external influence alter the oscillation of the FeCNT. Through the co-resonant coupling this changes the resonance frequencies of the coupled system and can be detected at the cantilever.
3. Small interactions result in rather large frequency shifts due to the low stiffness, i.e., high sensitivity of the nanoscale oscillator, and can be measured with a rather insensitive cantilever.

### Sensor fabrication

**Figure 3:**
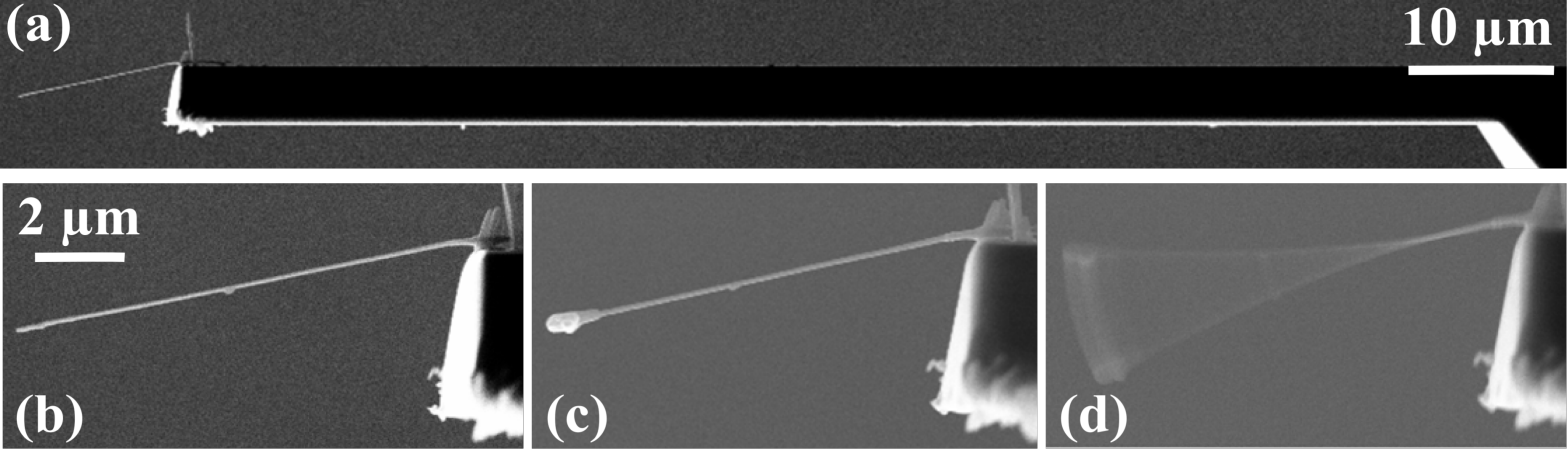
SEM images (a) of the fabricated sensor, (b) and (c) of the free end of the FeCNT before and after carbon deposition and (d) of the co-resonant oscillation of the FeCNT.

Based on the theoretical considerations we fabricated a magnetometry sensor consisting of a commercially available tipless silicon cantilever and an iron-filled carbon nanotube. All productions steps were carried out in a Zeiss FIB CrossBeam 1540 XB under high vacuum (≈10^−5^ mbar). First, the cantilever was shortened via focused ion beam milling to increase its resonance frequency. This step also increased the stiffness of the cantilever to about 133.8 N/m (see Table 1) which is rather high compared to typical values in cantilever magnetometry. In a second step, an individual nanotube was picked from a forest of FeCNTs grown by chemical vapor deposition [10] by a Kleindiek micromanipulator and placed at the free end of the cantilever. Electron beam-induced deposition of amorphous carbon on the contact point between FeCNT and cantilever ensures a strong attachment of the nanotube. Next, amorphous carbon was also deposited at the free end of the FeCNT to lower its resonance frequency from above 2 MHz close to that of the cantilever. Throughout the process of carbon deposition, the oscillation of the FeCNT was observed with SEM to ensure close matching of the two resonance frequencies by employing a custom-made vibration stage. When the resonance frequencies of the subsystem are approaching each other, the resonance frequencies of the coupled system do not coincide with them anymore, as discussed above. In order to still measure the single resonance frequency of each subsystem we used a tungsten tip to hold the respective other subsystem, therefore detuning the coupled system. Figure 3 shows the sensor and magnified images of the free of the FeCNT end before and after frequency matching. Furthermore, the co-resonant oscillation of the nanotube is depicted.

Another feature of the co-resonant coupling is the amplitude amplification [13] of the free end of the oscillating nanotube as it is evident in Figure 3d. The free end of the FeCNT exhibits an amplitude of more than 2 μm, whereas the cantilever and the other end of the FeCNT conntected to the cantilever only oscillate in the range of a few nanometers.

### Magnetometry measurement

All magnetometry measurements were carried out in a NanoScan AG hr-MFM at room temperature and under high vacuum (≈10^−5^ mbar). The machine employs a piezo actuator for oscillating the cantilever and a laser deflection detection system with a sectioned photo diode to determine its oscillation. We measured the amplitude response of the cantilever by sweeping the excitation frequency at a constant amplitude of the AC piezo voltage.

**Figure 4:**
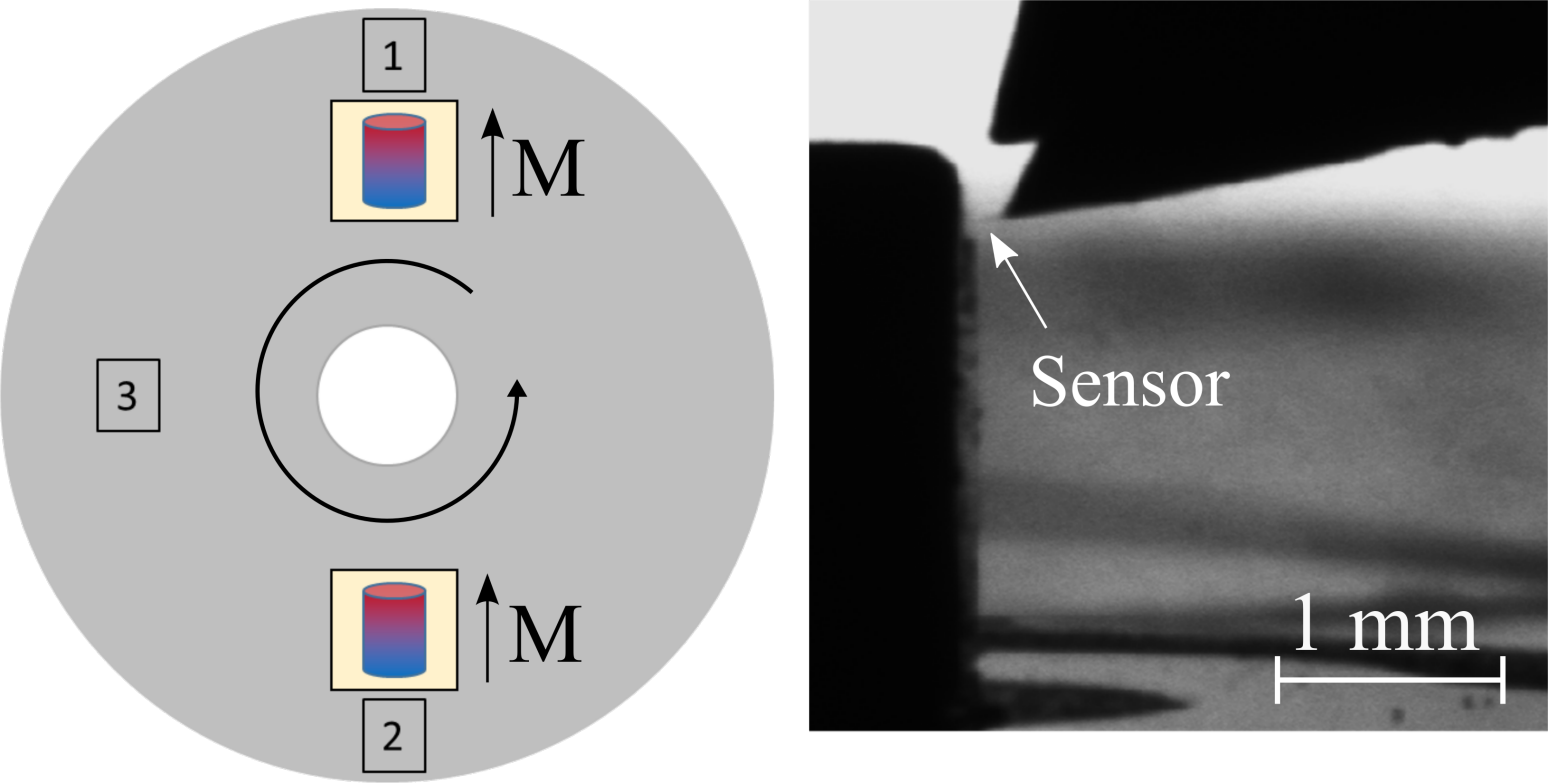
Sketch of measurement positions which are reached by keeping the sensor position fixed and rotating the sample plate. (1) and (2) correspond to the two orientations of the magnetic field and (3) is a field-free position. The CCD camera picture on the right hand side shows the sensor close to one of the magnets.

In order to generate a magnetic field parallel to the long axis of the FeCNT inside the measurement chamber, we used commercially available NdFeB magnets [14], positioned on a sample plate. The sensor position was fixed throughout the measurement and the sample plate carrying the magnets has been rotated in order to allow measurements with both magnetic field directions as well as at a field-free position. Figure 4 depicts a sketch of the setup with the measurement positions indicated and an image generated by the CCD camera of the instrument.

**Figure 5:**
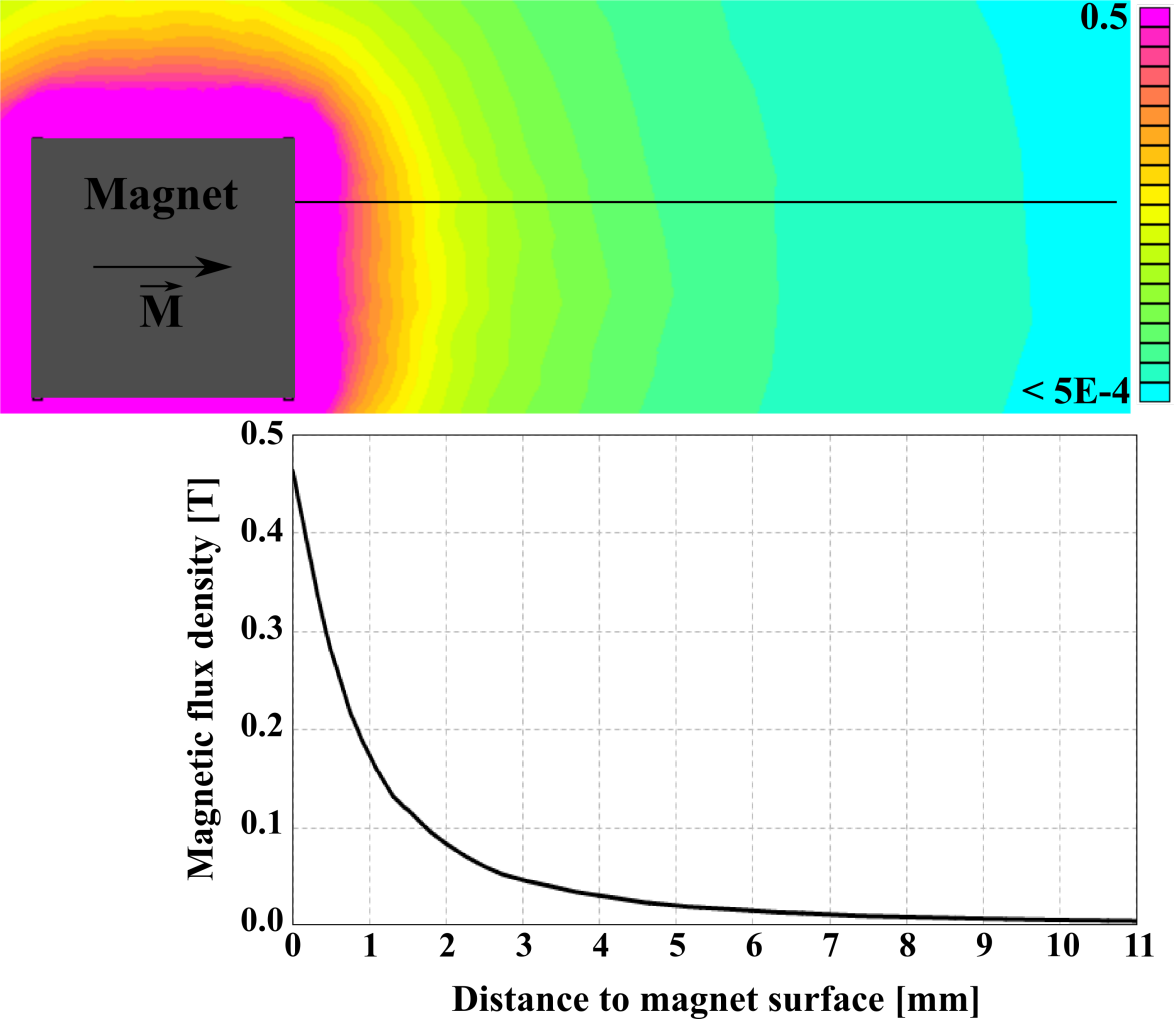
Simulated magnetic field of the permanent magnet. The field dependence on the distance to the surface of the magnet is calculated for the black line, corresponding to the measurement height.

Furthermore, Figure 5 shows a two-dimensional simulation of the magnetic field obtained with the finite element software FEMM [15]. Close to the surface of the magnet at the height, where the measurements were carried out, the magnetic flux density reaches values of about 460 mT in the direction of the FeCNT’s easy axis.

**Figure 6:**
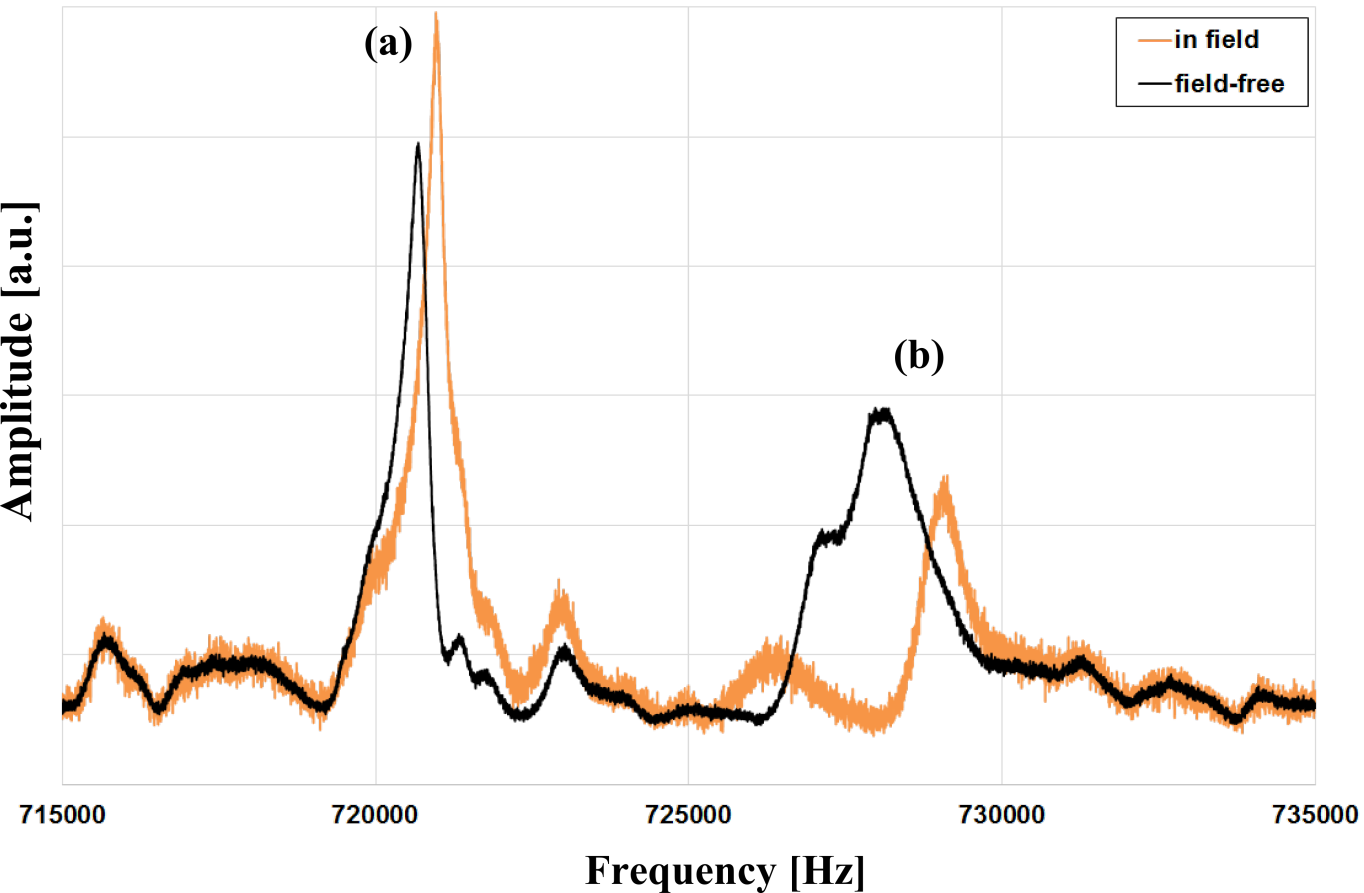
Amplitude response curves of the cantilever measured at the field-free position (3) according to Figure 4 and approximately 100 μm above the surface of the magnet (position (2)). The peaks are marked with (a) and (b) for reference purposes.

In order to measure at various magnetic field values without moving the sensor to ensure stable measurement conditions, the distance between sensor and magnet was changed by stepwise movement of the sample plate holding the magnet. At each step we measured the amplitude response of the cantilever in the frequency range of interest and determined the two resonance frequencies of the coupled system. Figure 6 shows two amplitude response curves as an example: one being taken at the position closest to the magnet, i.e., at a distance of about 100 μm in a field of approximately 406 mT, and the other one at a field-free position (see Figure 4). As can easily be seen, there is a significant change in the resonance frequencies of the coupled system which we attribute to the magnetic interaction between the iron filling of the FeCNT and the magnetic field of the NdFeB magnet.

**Figure 7:**
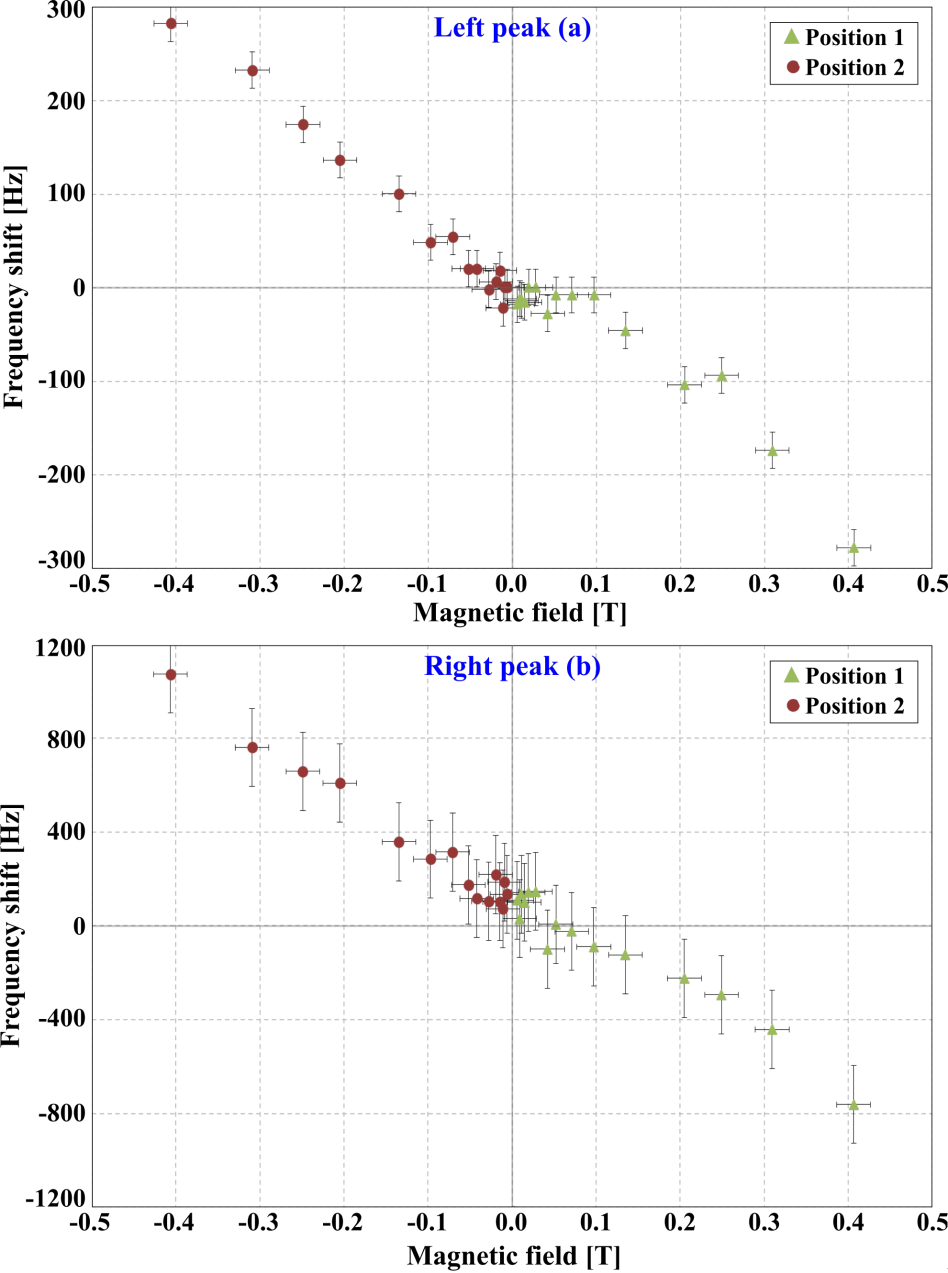
Measured frequency shifts of both peaks (a) and (b) compared to the field free measurement for various magnetic field values.

Figure 7 depicts the measured resonance frequency shift for each of the two peaks of the coupled system compared to the field free measurement for various magnetic field values. We observe a frequency shift of several 100 Hz in high magnetic field compared to the field free measurement for the left peak (a) in Figure 6. The shifts are even higher for the smaller, i.e., the right hand side, peak but it also features a higher measurement uncertainty regarding the determination of the maximum amplitude and hence resonance frequency. Table 2 summarizes the maximum frequency shift for both peaks and orientations of the magnetic field. The differences in the frequency shift values can be attributed to limited position accuracy of the magnets.

**Table 2:** Measured resonance frequencies *f*_a/b_ of the coupled system for both orientations of the magnet and corresponding frequency shift values Δ*f* compared to a field-free measurement. The values are given for the highest magnetic field.

	Left peak (a)	Right peak (b)

*f*_a/b_ field-free	(720679.5 ± 10) Hz	(728051.3 ± 83) Hz
Position 1		
*f*_a/b_ @ 406 mT	(720402 ± 10) Hz	(727290 ± 83) Hz
Δ*f*	(−277.5 ± 20) Hz	(−761.3 ± 166) Hz
Position 2		
*f*_a/b_ @ −406 mT	(720962 ± 10) Hz	(729124 ± 83) Hz
Δ*f*	(282.5 ± 20) Hz	(1072.7 ± 166) Hz

## Results and Discussion

Compared to previous cantilever magnetometry experiments with similar FeCNTs, the frequency shifts in our experiment of 280 Hz and 1000 Hz, respectively, for the two resonance peaks are increased by several orders of magnitude. For example, Banerjee et al. used similar FeCNTs on sensitive cantilevers with spring constants of about 0.2 N/m at low temperatures and measured frequency shifts in the order of mHz [7]. It has also been shown previously that a FeCNT oscillating in a magnetic field without being placed onto a cantilever can indeed exhibit a large frequency shift compared to the field-free case. However, the detection of the oscillatory state of the small nanotube remained a challenge as stated by Philippi et al. [16]. With our approach of co-resonant coupling we simultaneously allow for a very strong measurement signal and an easy detection of the oscillatory state of the FeCNT.

After the rather qualitative analysis of our obtained data, we now want to show the possibility to extract reliable magnetic information from the measured frequency shifts, making our sensor suitable for quantitative magnetic measurements.

### Effective spring constant of the coupled system

In order to derive magnetic information from the measured frequency shift data, we use a relation similar to Equation 2 by introducing effective spring constants 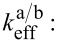


[4]
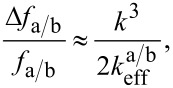


and evaluate it with respect to *k*_3_ which contains the magnetic interaction. Here *f*_a/b_ and Δ*f*_a/b_ denote the two resonance frequencies of the coupled system and their respective shifts. The effective spring constants 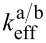 determine the sensitivity for each resonance peak of the coupled system and have to be known for a quantitative analysis of the measured data.

In order to obtain them for the given sensor geometry we will be using an approximate formula to calculate the expected resonance angular frequencies of the coupled system ω_a/b_ = 2π*f*_a/b_ for a small interaction spring constant *k*_3_[17]:

[5]
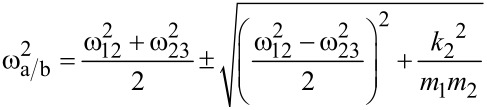


with 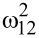 = (*k*_1_ + *k*_2_)/*m*_1_ and 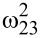 = (*k*_2_ + *k*_3_)/*m*_2_. Furthermore, *m_1,2_* denote the effective masses of the subsystems which can easily be determined from their resonance frequencies (see Table 1) by *m_1,2_* = *k_1,2_*/(2π*f_1,2_*)^2^. Equation 5 neglects any damping effects but this is a justified approximation since all our measurements are carried out under high vacuum, limiting damping to intrinsic effects due to the bending of the oscillating structures [9]. This was futhermore confirmed by comparison between the results obtained by Equation 5 and evaluating of the analytical solution of the differential equations describing the system.

With Equation 5 we can calculate the resonance frequencies of the coupled system for the two cases: without interaction, i.e., *k*_3_ = 0, and with a small interaction, *k*_3_ = 1 · 10^−6^ N/m, and determine the resulting frequency shifts. By inserting these values in Equation 4 and rearranging it, we are able to obtain the values for the effective spring constants for the two resonance peaks as:


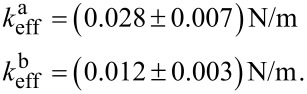


Comparing these values to the spring constants of the single subsystems *k*_cant_ = 133.8 N/m and *k*_CNT_ = 0.0086 N/m it is obvious that the effective spring constants of the coupled system are a mixture of the stiffnesses of the individual subsystems and that they are strongly influenced by the low stiffness of the FeCNT. Furthermore, the effective spring constants depend on the grade of frequency matching as can be seen from Equation 5. Closely matched resonance frequencies induce a strong interplay between the two subsystems. Hence, any magnetic interaction between FeCNT and external influences can be sensed with an effective stiffness slightly above the low spring constant of the FeCNT (in our case approximately by a factor of three) but be measured with a rather insensitive cantilever. However, we do not see the full sensitivity of the FeCNT in the measured frequency shift. Instead we observe a reduction leading to the conclusion that the behaviour of the coupled system can be described well by the effective spring constant *k*_eff_.

**Figure 8:**
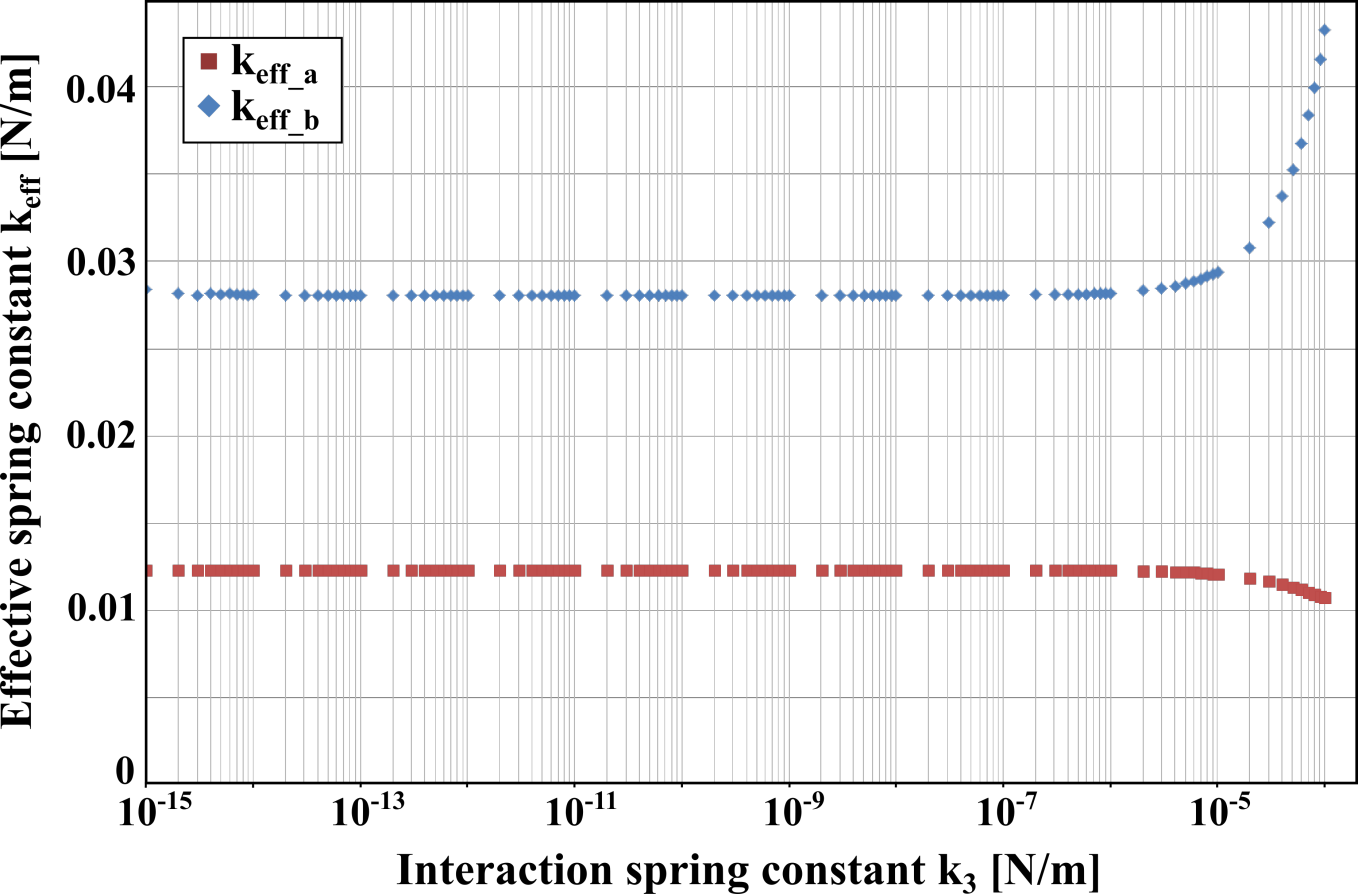
Dependence of the effective spring constant of each peak 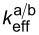 on the interaction spring constant *k*_3_. The calculations are based on the properties of the system given in Table 1.

However, it is important to note that *k*_eff_ is only constant as long as *k*_3_*<<k*_1_; *k*_2_ is satisfied. Otherwise it shows a strong dependence on *k*_3_. Calculations for our set of parameters depicted in Figure 8 indicate that *k*_3_ has to be at least two orders of magnitude below the smaller spring constant of the system. This is well fulfilled in our case since the interaction spring constant for the strongest magnetic field of |*B*| = 406 mT is approximately 2 · 10^−5^ N/m (calculated with Equation 4). Please note that the results in Figure 8 are only shown for *k*_3_ ¿ 0 but the behaviour is approximately similar for *k*_3_*<* 0, only with the deviations from the constant values being in the opposite direction.

Since for closely matched frequencies *k*_eff_ is mainly dominated by the smaller subsystem, a decrease of its stiffness will lead to an increase in sensitivity. Hence, possible sensor implementations could include the use of single walled carbon nanotubes as smaller oscillator or fabrication of the complete sensor in silicon technology, allowing for production of double cantilever structures with one nanocantilever [13,18].

However, it has to be noted that the low effective spring constant is not the sole reason for the increased signal strength of the proposed sensor setup. Other groups employed low-stiffness cantilevers for magnetometry measurements as well but did not obtain such a strong frequency shift signal. In cantilever magnetometry the signal is related to the length of the sensing cantilever and the spring constant as stated by Equation 3. Therefore, an increased cantilever length would in principle favorably affect the spring constant but nevertheless limits the sensitivity increase. In view of that our geometry is favorable as well since the sensing part, i.e., the nanotube, has a low spring constant while also being relatively short (in the order of 10 μm). Both features contribute to the observed strong increase in signal strength.

### Determination of effective magnetic monopole moment from measured data

With the determination of *k*_eff_ it is possible to extract magnetic information from the measured frequency shift data, i.e., *k*_3_. It has been shown that in case of a FeCNT a suitable magnetic quantity is the effective magnetic monopole moment *q* of the iron nanowire [19–20]. Due to its single-domain magnetic structure it is acting as an elongated magnetic dipole oscillating in a magnetic field. Since the magnetization of the iron nanowire is considered to be nearly parallel to the axis of the FeCNT, its two magnetic poles are positioned at either end, i.e., at a distance of *L*_cnt_ = 10 μm. Furthermore, a decrease of the field of the NdFeB magnet along the length of the FeCNT of maximal 5 mT is assumed. The interaction of each of the two poles with the external magnetic field leads to a contribution to the measured frequency shifts Δ*f*_a/b_. However, the magnetic pole at the attachement point between nanotube and cantilever gives a much lower contribution for several reasons: its oscillation trajectory radius and sensor stiffness are mainly given by the cantilever. In contrast to that the sensor stiffness at the free end of the FeCNT can be described by the soft effective spring constant of the coupled system. Hence, only the monopole at the free end of the FeCNT contributes to the frequency shift and the influence of the other pole can be neglected. Thus, the effective magnetic monopole moment proves to be a suitable parameter to characterize the magnetostatic behaviour of the iron of the FeCNT filling in the low external field approximation.

Following Philippi et al. [16], the effective magnetic monopole moment *q* can be related to the interaction spring constant *k*_3_:

[6]
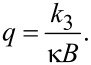


Here *B* is the magnetic flux density generated by the NdFeB magnet at the position of the free end of the FeCNT (see Figure 5). The parameter κ is given as the curvature of the oscillating termination point of the FeCNT and has been obtained from SEM pictures. The mechanical behaviour of an oscillating nanowire is discussed in depth elsewhere [16] and for our sensor we determined κ = (0.16 ± 0.016) μm^−1^.

By inserting Equation 6 in Equation 4 we obtain the relation:

[7]
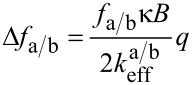


which is used for a linear regression analysis of the measured frequency shift data in Figure 7 using the low field range |*B*| *<* 0.25 T to avoid major deviations from the monopole approach. This evaluation yields two *q*-values (*q*_1_ = 3.05 · 10^−10^ A·m and *q*_2_ = 4.37 · 10^−10^ A·m) corresponding to the two resonance peaks of the coupled system. Since the effective magnetic monopole moment has to be the same for both peaks we calculate the mean value and finally obtain *q* = (3.7 ± 0.7) · 10^−10^ A·m, which corresponds to a magnetic moment of approximately 4 · 10^8^μ_B_ (CNT length 10 μm).

### Geometric effective magnetic monopole moment

For comparison, the *q*-value of the FeCNT can be determined from geometric information obtained from high-resolution SEM pictures. With a diameter of the iron filling of the FeCNT of *d*_Fe_ = (22 ± 6) nm and saturation magnetization of iron *M*_s,Fe_ = (1.71 ± 0.01) · 10^6^ A/m, the corresponding effective monopole moment is:

[8]
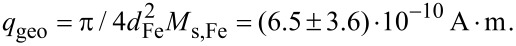


Despite the relatively large margin of uncertainty due to the diameter measurement of the iron filling, this result corresponds well to the effective magnetic monopole moment determined from the frequency shift values. Furthermore, we can also compare our results to values obtained in other experiments with iron-filled carbon nanotubes of similar size and find a good agreement [7,16,21]. These results demonstrate that the co-resonant sensor concept applied to cantilever magnetometry allows for a quantitative determination of magnetic sample properties with strongly increased frequency shifts compared to single-cantilever magnetometry experiments [7].

### Measurement limits

When proposing a novel sensor concept, inevitably the question regarding the measurement limit arises. The sensitivity limits of cantilever-based magnetometry sensors have already been discussed by various groups [3,22–23], leading to three main considerations which have to be taken into account: the sensitivity of the detection setup and thermal and magnetic noise in the oscillating system. From these, thermal noise is considered to be the most dominant one [24], followed by the detector noise which is statistically independent of the former [25]. For our discussion, we will only focus on thermal noise. Even if only this single noise source is considered it is still an open question how the noise is distributed in the co-resonantly coupled sensor system [26]. We will therefore use the approach of discussing the noise limits for each subsystem, indicating a range of the sensitivity for the coupled system.

In standard cantilever magnetometry the noise limits the detectable frequency shift and, hence, the minimal detectable magnetic moment. The thermal limit for the frequency shift for a cantilever is given by [25]:

[9]
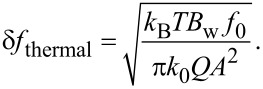


Hereby *k*_B_ denotes the Boltzmann constant, *T* the temperature, *B*_w_ the measurement bandwidth and *Q* the quality factor. *f*_0_ and *k*_0_ are the resonance frequency and spring constant of the cantilever and *A* is the amplitude at the free end of the cantilever. By following the reasoning of Gysin et al. [23], the frequency shift Δ*f* induced by a high-aspect ratio, single domain ferromagnetic cylinder placed on a cantilever is given by Equation 3. Combining Equation 3 and Equation 9, the minimal detectable magnetic moment is:

[10]
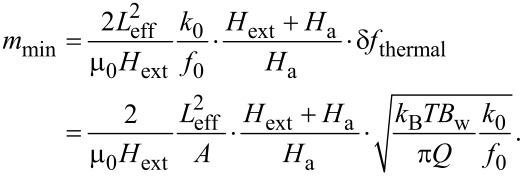


From this expression one can conclude that it is favorable to have a small cantilever length, a high oscillation amplitude, a strong magnetic field, small spring constant, high resonance frequency and a large quality factor. Both cantilever length and amplitude have a major influence on the minimal detectable magnetic moment. Furthermore, *m*_min_ is maximized if *H*_ext_ and *H*_a_ are high.

The above expression has been derived for a single cantilever and it is still under investigation if and how it can be adapted for the coupled system. It is a reasonable assumption that, in accordance with the considerations of the effective spring constant, the quality factor will as well be an effective one for the coupled system. Similarly, the effective magnetic moment sensitivity of the coupled system is expected to lie in between the sensitivities of the two single systems. Therefore, in Table 3 we calculated the minimal detectable magnetic moment for both of our subsystems at room temperature, an external magnetic field of 1 T and a measurement bandwidth *B*_w_ = 1 Hz, in accordance with literature values for such calculations [3,23]. The necessary parameters for both subsystems are taken from Table 1 for the unmatched frequency state and the length of cantilever and nanotube have been taken from SEM pictures: *L*_cant_ = 85 μm and *L*_cnt_ = 10 μm. They have been recalculated into effective lengths by *L*_eff_ = *L*/1.377 for the first bending mode [6]. We are aware of the fact that there are carbon nanotubes, especially single wall CNTs, that can exhibit a much lower stiffness than the FeCNT presented here but we want to stick to our existing experimental system and have taken the mechanical properties of the CNT as measured.

**Table 3:** Minimal detectable magnetic moment for both subsystems of the coupled system. The calculations have been made for room temperature *T* = 293 K, a bandwidth of *B*_w_ = 1 Hz and magnetic field of *B*_ext_ = 1 T.

Parameter	Cantilever	FeCNT

spring constant [N/m]	133.8	0.0086
amplitude [nm]	10	1000
δ*f* [mHz]	5	26
length [μm]	85	10
*m*_min_ [A·m^2^]	6 · 10^−15^	1 · 10^−20^
*m*_min_ / μ_B_	7 · 10^8^	1 · 10^3^

From Table 3 we see that the cantilever has a lower minimal detectable frequency shift (due to its better *Q*-factor and smaller resonance frequency) compared to the nanotube but still the nanotube exhibits a much better magnetic moment resolution of 10^3^ μ_B_ at room temperature. It has to be kept in mind that the magnetization of small particles might not be thermally stable.

Comparing these calculated values to the measured magnetic moment of the section ’Determination of effective magnetic monopole moment from measured data’, which was *m*_FeCNT_ ≈ 4 · 10^8^ μ_B_, we see that it is already at the limit of what could be measured with only the silicon cantilever. In our experiment, we observed a frequency shift of several 100 Hertz for the resonance frequencies of the coupled system which already indicates that the magnetic moment sensitivity is significantly increased compared to that of a single cantilever. Extending the considerations of Table 3 to single-wall carbon nanotubes and applying low temperatures, a potential moment sensitivity in the order of single Bohr magnetons could be achieved. However, as for the effective spring constant, it will probably not be possible to obtain the full moment sensitivity of the nanotube due to the strong interplay between the subsystems, but by using softer cantilevers and softer (unfilled) CNTs the coupled sensor concept has a great potential of achieving very low magnetic moment resolution while maintaining an easy detection at the same time.

## Conclusion

We applied the universal concept of a co-resonantly coupled sensor to cantilever magnetometry by using a commercially available silicon cantilever and an iron-filled carbon nanotube. The FeCNT acted simultaneously as nanocantilever and magnetic sample and, since the magnetic properties of similar FeCNTs had already been studied, allowed for demonstrating the functionality of the sensor setup. The evaluation of the measurements shows that, once calibrated by determining the effective spring constant, the sensor can be used to derive magnetic properties of nanometer-sized samples and significantly increases the signal strength by several orders of magnitude compared to conventional single-cantilever magnetometry experiments. A further increase is possible by tailoring the components of the coupled system according to the measurement task. The basic principles of the co-resonant sensor concept [9] are of general nature and therefore not limited to cantilever magnetometry but can also be used to fabricate mass and force sensors in general.
